# Free-Energy and Molecular Dynamics Analysis of Ser195 and Thr195 Rotameric States in Thrombin

**DOI:** 10.64898/2026.10.13.758922

**Published:** 2026-10-18

**Authors:** Stephanie M. Sauve, Parth Chaturvedi, Mahmoud Moradi

## Abstract

Ser195 is highly conserved as the catalytic nucleophile in trypsin-like serine proteases, yet the molecular basis for the strong preference for serine over the chemically similar threonine residue remains unclear. Here, we used well-tempered metadynamics and microsecond-long molecular dynamics (MD) simulations to characterize the conformational energetics of S195 and T195 in thrombin. Well-tempered metadynamics revealed distinct rotameric free-energy landscapes in both the apo and the ligand-bound systems. In the apo state, S195 preferentially occupied a lower-angle rotameric state, while T195 exhibited two low-energy states with similar free energies. Unbiased simulations further showed greater conformational heterogeneity and increased higher-angle sampling for T195. Evaluation of the His57–residue 195 hydrogen bond further revealed that S195 maintained close hydrogen bond distances across both rotameric regions, whereas T195 exhibited longer and more variable His57–hydroxyl distances with reduced distance-based occupancy. Analysis of the local environment showed that the additional T195 methyl group was associated with redistributed protein contacts. Additional rotamer-conditioned analyses demonstrated differences in local packing within corresponding conformational states. Furthermore, solvent accessibility and water-contact analyses revealed that the effects of the substitution on the catalytic side-chain environment depend on rotameric conformation. Together, these findings demonstrate that two complementary simulation approaches converge on similar conformational preferences and suggest that the S195T substitution alters the rotameric free-energy landscape in association with changes in catalytic geometry, local packing, and solvation. These results provide insight into the evolutionary preference for serine over threonine at the catalytic position of thrombin.

